# Enrichment of the Postdischarge GRACE Score With Deceleration Capacity Enhances the Prediction Accuracy of the Long-Term Prognosis After Acute Coronary Syndrome

**DOI:** 10.3389/fcvm.2022.888753

**Published:** 2022-04-27

**Authors:** Shoupeng Duan, Jun Wang, Fu Yu, Lingpeng Song, Chengzhe Liu, Ji Sun, Qiang Deng, Yijun Wang, Zhen Zhou, Fuding Guo, Liping Zhou, Yueyi Wang, Wuping Tan, Hong Jiang, Lilei Yu

**Affiliations:** ^1^Department of Cardiology, Renmin Hospital of Wuhan University, Wuhan, China; ^2^Cardiac Autonomic Nervous System Research Centre of Wuhan University, Wuhan, China; ^3^Cardiovascular Research Institute, Wuhan University, Wuhan, China; ^4^Hubei Key Laboratory of Cardiology, Wuhan, China

**Keywords:** acute coronary syndrome, GRACE score, deceleration capacity, autonomic nerve, long-term prognosis

## Abstract

**Background:**

Cardiac autonomic nerve imbalance has been well documented to provide a critical foundation for the development of acute coronary syndrome (ACS) but is not included in the postdischarge GRACE score. We investigated whether capturing cardiac autonomic nervous system (ANS)-related modulations by 24-h deceleration capacity (DC) could improve the capability of existing prognostic models, including the postdischarge Global Registry of Acute Coronary Events (GRACE) score, to predict prognosis after ACS.

**Method:**

Patients with ACS were assessed with 24-h Holter monitoring in our department from June 2017 through June 2019. The GRACE score was calculated for postdischarge 6-month mortality. The patients were followed longitudinally for the incidence of major adverse cardiac events (MACEs), set as a composite of non-fatal myocardial infarction and death. To evaluate the improvement in its discriminative and reclassification capabilities, the GRACE score with DC model was compared with a model using the GRACE score only, using area under the receiver-operator characteristic curve (AUC), Akaike's information criteria, the likelihood ratio test, category-free integrated discrimination index (IDI) and continuous net reclassification improvement (NRI).

**Results:**

Overall, 323 patients were enrolled consecutively. After the follow-up period (mean, 43.78 months), 41 patients were found to have developed MACEs, which were more frequent among patients with DC <2.5 ms. DC adjusted for the GRACE score independently predicted the occurrence of MACEs with an adjusted hazard ratio (HR) of 0.885 and 95% CI of 0.831–0.943 (*p* < 0.001). Moreover, adding DC to the GRACE score only model increased the discriminatory ability for MACEs, as indicated by the likelihood ratio test (χ^2^ = 9.277, 1 df; *p* < 0.001). The model including the GRACE score combined with DC yielded a lower corrected Akaike's information criterion compared to that with the GRACE score alone. Incorporation of the DC into the existing model that uses the GRACE score enriched the net reclassification indices (NRIe^>0^ 7.3%, NRIne^>0^ 12.8%, NRI^>0^ 0.200; *p* = 0.003). Entering the DC into the GRACE score model enhanced discrimination (IDI of 1.04%, *p* < 0.001).

**Conclusion:**

DC serves as an independent and effective predictor of long-term adverse outcomes after ACS. Integration of DC and the postdischarge GRACE score significantly enhanced the discriminatory capability and precision in the prediction of poor long-term follow-up prognosis.

## Introduction

Acute coronary syndrome (ACS) occurs in people who are prone to exacerbations and adverse outcomes, and optimizing the risk stratification of these patients is of considerable clinical interest (1, 2). Therefore, even if the optimal treatment is known, identifying which ACS patients will benefit the most from early interventional treatment can be challenging. Currently, the Global Registry of Acute Coronary Events (GRACE) score is the most well-established risk stratification tool for the prediction of in-hospital and long-term mortality and the risk of myocardial infarction after ACS (3, 4). Although it has been well established that the overall prognosis after ACS is worse among those with cardiac autonomic nerve imbalance than among those without cardiac autonomic nerve imbalance (5–8), the GRACE score does not include data from wearable devices that test cardiac autonomic function.

Notably, the value of 24-h deceleration capacity (DC), a feasible and non-invasive indicator that captures autonomic activity-related modulations of heart rate, adds valuable and repeatable information for timely identification of ACS patients at higher risk and aid in risk stratification (5–8). Accordingly, previous studies have also demonstrated that DC may serve as a predictor of mortality and outperformed a standard measure of heart rate variability (HRV) (8). Moreover, adjustment of the admission GRACE score, calculated for the prediction of in-hospital mortality, by short-term DC improves the accuracy of prediction of the composite of mortality, including in-hospital, 30 and 180-day mortality, among patients with suspected ACS (9). However, whether the readily accessible clinical 24-h DC remains a significant prognostic factor to enhance the predictability of prognostic models, including the postdischarge GRACE score, for ACS patients after long-term follow-up remains unclear.

Therefore, we investigated the value in long-term prognosis of 24-h DC added to the postdischarge GRACE score among ACS patients and the underlying incremental prognostic value of entering DC into an existing model including the postdischarge GRACE score only.

## Methods

### Patient Population

**Figure 1:**
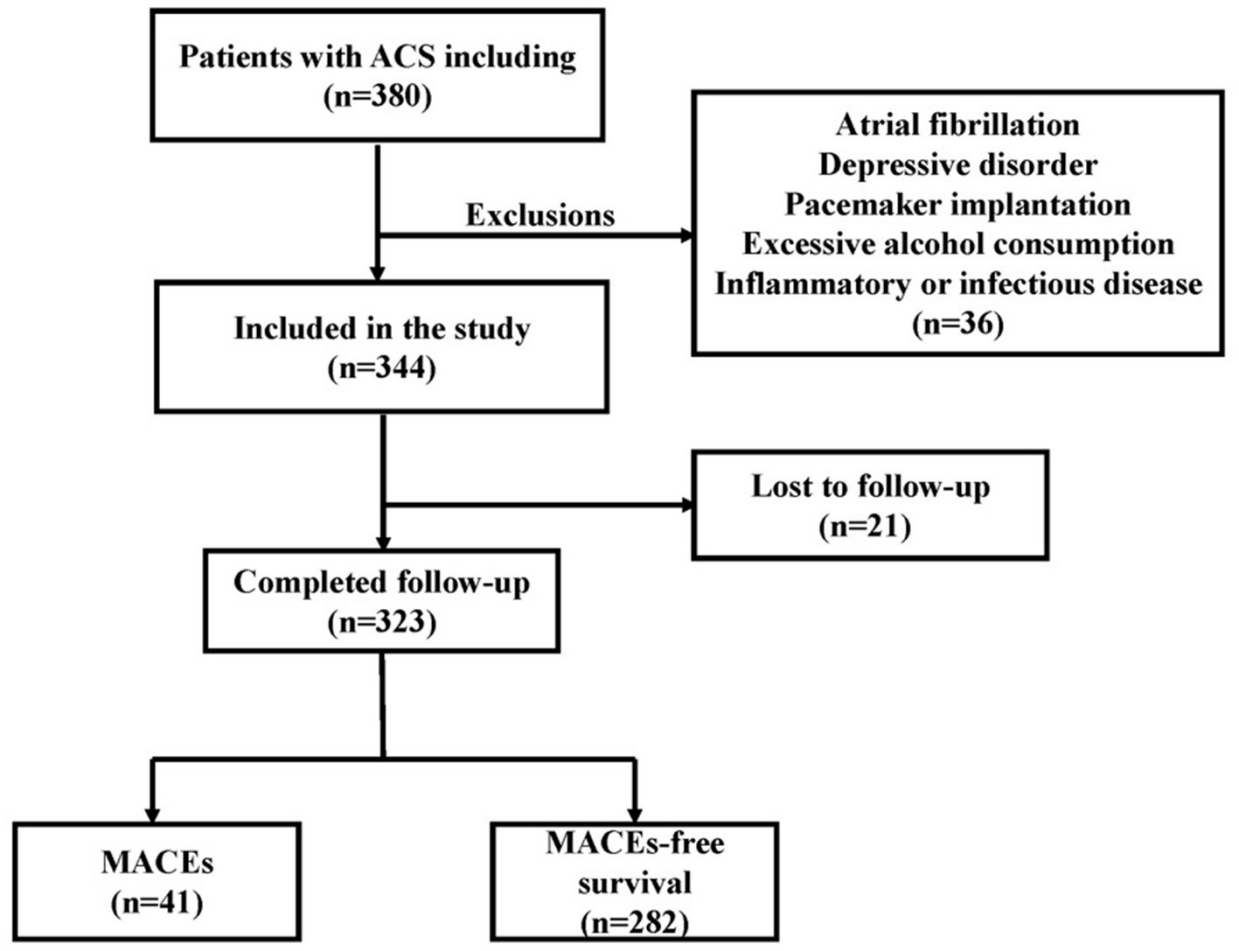
Flowchart of patient enrollment.

We retrospectively enrolled 323 consecutive patients with ACS at Renmin Hospital of Wuhan University from June 2017 through June 2019. The previously established guidelines addressed the process and criteria for acute coronary syndrome diagnosis (10). ACS included non-ST-elevation ACS (NSTE-ACS) and ST-elevation ACS (STE-ACS). Patients with NSTE-ACS included those presenting with unstable angina (UA) and non-ST-segment elevation myocardial infarction (non-STEMI), and patients with STE-ACS included those presenting with ST-elevation myocardial infarction (STEMI). The main exclusion criteria were as follows: atrial fibrillation, pacemaker implantation, use of any medications that affect heart rate, severe liver or renal insufficiency <30 ml/(min 1.73 m^2^), inflammatory or infectious disease, depressive disorder, hyperthyroidism and excessive alcohol consumption. The flowchart of participant enrollment is presented in Figure 1. Due to the purely retrospective observational, our study was exempt from requiring ethics approval and informed consent from eligible patients by the Renmin Hospital of Wuhan University Ethics Committee.

### Laboratory Examinations

Venous blood specimens were collected when patients were initially referred to the emergency department or cardiology department. All participants were subjected to routine whole blood analysis, including routine blood, blood glucose, kidney function, and plasma lipid analyses.

### Holter Monitoring and DC Analysis

All participants included in this study underwent 24-h Holter monitoring (DMS300-4A, DM Software, Inc., USA) recordings after coronary angiography. The 24-h mean heart rate, time-domain parameters [i.e., standard deviation of all normal sinus RR intervals (SDNN), standard deviation average of normal-to-normal (NN) intervals (SDANN), percentage of the number of times that the difference between adjacent normal RR intervals >50 ms in the total number of NN intervals (pNN50), root mean square successive difference (rMSSD)], frequency-domain parameters [i.e., high-frequency power (HF), low-frequency power (LF), and low-frequency/high-frequency ratio (LF/HF)] and DC were automatically specifically calculated via commercial software (H-Scribe Analysis System, Mortara Instrument, Inc., Milwaukee, WI, USA) as shown in a previous reference (11, 12). Frequency domain parameters yield a more accurate and detailed quantification of heart rate than time domain parameters (13, 14). Morevore, the predictive value of LF/HF may be superior to other parameters of HRV (15–17). DC analysis is divided into three steps. First, the RR scatter plot shows the scatter of the full range of beat-to-beat RR intervals, from which the starting time for analysis is selected. The analysis length and number of cardiac cycles were then chosen, with the analysis length generally defaulting to a full 24 h. Finally, the X (0), X (1), X (- 1), and X (- 2) values reflected in the heart rate deceleration curve were substituted into the formula DC = [x (0) + X (1) + X (- 1) + X (- 2)]/4 to compute the 24 h DC, and the resulting unit was ms.

### GRACE Score

The GRACE score was calculated at discharge to predict 6-month mortality (https://www.outcomes-umassmed.org/grace). The parameters of the GRACE score include age, heart rate, systolic blood pressure on arrival, creatinine level, percutaneous transluminal coronary intervention (PCI) during in-hospital period, coronary artery bypass grafting (CABG) during in-hospital period, previous myocardial infarction (MI), ST-segment depression, increased levels of cardiac enzyme/marker and congestive heart failure.

### Follow-Up

The average time to follow-up was 43.78 months. Patients were discharged, and follow-up was conducted through an outpatient follow-up or telephone follow-up. At the end of the follow-up, a total of 21 cases (6.1%) were lost, and 323 patients (93.9%) were followed to the end. The clinical endpoint of our study was a composite endpoint clinical events of major adverse cardiovascular events (MACEs), including death and non-fatal myocardial infarction. Two experienced physicians adjudicated the endpoint events according to medical record reviewing.

### Statistical Analysis

Continuous variables are represented by a mean plus a standard deviation (SD) or as the median with interquartile range (IQR) determined by skewness, whereas categorical variables are summarized as frequencies and percentages. All participants were stratified into three groups based on their DC [low-risk group (DC > 4.5 ms), intermediate-risk group (DC > 2.5 ms, and DC ≤ 4.5 ms), and high-risk group (DC ≤ 2.5 ms)]. Differences between groups were analyzed with one-way analysis of variance (ANOVA), the Mann–Whitney U test or the Kruskal–Wallis test depending on the normality of the distribution. Categorical variables were analyzed with the chi square (χ^2^) test. Survival free from MACEs was analyzed by the Kaplan–Meier method. Final covariates were age, sex, past history and laboratory results according to the results of the pre-survey. We used univariate Cox regression analyses first performed to determine the potential predictors of MACEs, followed by multivariate Cox analyses of significant variables with a *p*-value < 0.05 to improve the accuracy of the conclusions. Differences were considered statistically significant at *p* < 0.05. Statistical analysis was performed with SPSS software (version 23; SPSS).

The predictability of MACEs using DC, GRACE score and GRACE score combine with DC by receiver operator characteristic (ROC) curve analysis. We compared whether adding DC to the GRACE score would enhance the discriminative and reclassification capabilities of the models. The fit of each nested model was compared via the χ^2^ likelihood ratio test to assess whether the logistic regression model that integrated DC and the postdischarge GRACE score supported a significantly better fit than the model including the GRACE score alone. Comparison of the nested and non-nested models, including the GRACE score or DC or LF/HF added to the GRACE score, was weighted by calculating the corrected Akaike's information criterion (AICc), delta-AICc (δ AICc), and Akaike weights (wi) to represent the probability that a given model was the best predictive model in the set (18).

Predicted probabilities (%) of MACEs were generated by logistic regression models using the GRACE score alone and the GRACE score combined with LF/HF or DC. The addition of DC and LF/HF to the existing models with the GRACE score was evaluated with the predicted probabilities of MACEs implementing multiple methods of improvement in discrimination: increase in the area under the receiver operating characteristic curve (AUC), category-free continuous net reclassification improvement (cNRI^>0^) and integrated discrimination improvement (IDI). Given the presence of missing specific predefined clinical risk thresholds for the models containing the GRACE score, categorical NRI was not employed. The net percentage of patients with the event of interest correctly assigned a higher predicted risk was defined as the event NRI (NRIe), and the net percentage of persons without the event of interest correctly assigned a lower predicted risk was defined as the non-event NRI (NRIne). Total NRI was defined as the sum of the net percentages of persons with and without the events of interest correctly assigned a different predicted risk. The IDI was equal to the enhancement in discrimination slope defined as the mean difference in predicted risks between those with and without events. The IDI was equal to the difference in the initial and updated models in the discrimination slope formed between the mean predicted probabilities (%) of patients with and without events.

## Results

### Clinical Baseline Characteristics

The baseline features of all of the enrolled ACS patients classified into three groups according to the DC value are presented in Table 1. Our results indicated that patients with lower DC (≤2.5 ms) were likely to be older (*p* < 0.001) and have non-ST-elevation ACS (NSTE-ACS) (*p* = 0.004); higher creatinine levels (*p* = 0.047), glucose levels (*p* = 0.032) and average heart rate (*p* < 0.001); lower esti mated glomerularfiltrationrate (eGFR) (*p* = 0.016), SDNN (*p* < 0.001), rMSSD (*p* < 0.001), Pnn50 (*p* < 0.001), LF (*p* < 0.001), HF (*p* < 0.001), and LF/HF (*p* = 0.031); a higher GRACE score (*p* < 0.001) and GRACE risk (*p* = 0.001); history of MI (*p* = 0.021); and increased creatine kinase-MB (CK-MB) levels (*p* = 0.034) and incidence of MACEs (*p* < 0.001).

**Table 1:** Baseline characteristics of the study population categorized by deceleration capacity (DC).

	**Low risk group** **(DC > 4.5 ms)** **(*n* = 183)**	**Intermediate risk group** **(DC > 2.5 ms, and DC ≤ 4.5 ms)** **(*n* = 94)**	**High risk group** **(DC ≤ 2.5 ms)** **(*n* = 46)**	**F/Z/χ2**	***P*** **-value**
Male, *n* (%)	114 (62.3)	57 (60.6)	35 (76.1)	3.592	0.166
Age (years)	61.43 ± 10.18	67.47 ± 9.21	65.13 ± 11.45	11.587	<0.001
Hypertension (%)	113 (61.7)	68 (72.3)	27 (58.7)	3.799	0.150
Duration of hypertension (years)	10.00 (5.00, 12.00)	10.00 (5.25, 18.75)	10.00 (3.00, 15.00)	2.596	0.273
Diabetes (%)	47 (25.7)	23 (24.5)	14 (30.4)	0.594	0.743
Duration of diabetes (years)	6.00 (2.00, 15.00)	5.00 (2.00, 10.00)	10.00 (2.00, 17.00)	0.727	0.695
Current smoker (%)	65 (35.5)	30 (31.9)	17 (37.0)	0.479	0.787
Duration of smoking (years)	10.00 (0.000, 20.00)	17.50 (4.50, 20.00)	10.00 (6.00, 17.50)	0.692	0.708
Current smoking cigarettes (per day)	28.00 (20.00, 30.00)	30.00 (17.25, 40.00)	30.00 (21.50, 40.00)	1.292	0.524
History of drinking (%)	42 (23.0)	14 (14.9)	7 (15.2)	3.196	0.202
Family history (%)	17 (9.3)	3 (3.2)	3 (6.5)	3.521	0.172
Previous PCI (%)	56 (30.6)	26 (27.7)	11 (23.9)	0.885	0.642
Clinical presentation				11.272	0.004
STEMI	115 (62.8)	43 (45.7)	19 (41.3)		
NSTE-ACS	68 (37.2)	51 (54.3)	27 (58.7)		
Neutrophil (× 10^9^/L)	3.98 (3.01, 4.86)	3.98 (3.16, 4.97)	4.24 (3.15, 5.71)	1.756	0.416
Lymphocyte (× 10^9^/L)	1.67 (1.32, 2.08)	1.52 (1.14, 1.92)	1.49 (1.21, 2.02)	4.969	0.083
NLR	2.29 (1.78, 3.20)	2.46 (1.88, 3.63)	2.63 (1.94, 4.45)	4.398	0.111
PLT (× 10^9^/L)	203.85 ± 55.28	205.61 ± 52.73	194.83 ± 60.48	0.628	0.535
PLR	121.88 (88.24, 156.99)	133.24 (95.97, 183.55)	122.32 (92.34, 162.58)	3.512	0.173
hs-CRP (mg/L)	0.90 (0.50, 3.85)	0.65 (0.50, 5.00)	1.97 (0.32, 5.68)	1.322	0.516
eGFR ml/(min1.73 m^2^)	91.13 ± 17.05	84.59 ± 17.28	88.43 ± 21.58	4.205	0.016
Creatinine (μmol/L)	69.00 (56.00, 81.00)	72.00 (57.75, 87.25)	75.00 (60.75, 91.00)	6.135	0.047
Uric acid (mmol/L)	375.26 ± 112.51	376.20 ± 114.29	402.57 ± 154.30	1.003	0.368
Glucose (mmol/L)	5.90 ± 2.21	6.46 ± 2.38	6.87 ± 3.59	3.473	0.032
TG (mmol/L)	1.48 (1.06, 1.99)	1.40 (1.08, 1.84)	1.15 (0.80, 1.73)	6.009	0.051
TC (mmol/L)	4.16 ± 1.10	4.24 ± 1.05	4.06 ± 0.94	0.440	0.644
HDL-C (mmol/L)	1.08 ± 0.29	1.06 ± 0.33	1.06 ± 0.27	0.065	0.937
LDL-C (mmol/L)	2.44 ± 1.00	2.44 ± 1.00	2.23 ± 0.84	0.873	0.419
Lp (a) (g/L)	152.00 (64.00, 298.00)	143.50 (67.00, 332.50)	134.00 (62.25, 295.25)	0.176	0.916
Average heart rate (bpm)	66.13 ± 7.41	72.89 ± 9.15	75.52 ± 12.63	30.948	<0.001
SDNN (ms)	119.00 (101.00, 137.00)	98.00 (79.00, 118.50)	97.00 (74.75, 120.00)	38.431	<0.001
SDANN (ms)	74.00 (50.00, 106.00)	77.00 (41.50, 100.00)	76.00 (53.00, 98.00)	0.837	0.658
rMSSD (ms)	31.00 (25.00, 46.00)	25.50 (19.00, 56.25)	17.00 (13.00, 27.75)	40.389	<0.001
Pnn50	6.00 (3.00, 12.00)	2.47 (0.14, 10.98)	1.00 (0.47, 1.99)	53.298	<0.001
LF (ms^2^)	286.00 (215.50, 459.10)	128.50 (81.68, 198.48)	101.50 (53.50, 160.18)	115.529	<0.001
HF (ms^2^)	202.00 (127.70, 294.00)	86.00 (46.75, 188.95)	71.10 (48.50, 225.00)	55.470	<0.001
LF/HF	1.50 (1.00, 2.37)	1.29 (0.80, 2.38)	1.30 (0.67, 1.81)	6.946	0.031
GRACE score	90.68 ± 23.73	105.77 ± 22.99	107.93 ± 32.06	16.121	<0.001
GRACE risk				18.142	0.001
High	52 (28.4)	9 (9.6)	5 (10.8)		
Intermediate	90 (49.2)	55 (58.5)	24 (52.2)		
Low	41 (22.4)	30 (31.9)	17 (37.0)		
SBP (mmHg)	133.65 ± 18.52	133.48 ± 21.12	131.79 ± 19.81	0.160	0.852
DBP (mmHg)	76.73 ± 11.63	76.31 ± 12.93	76.88 ± 12.39	0.048	0.953
History of MI (%)	22 (12.0)	13 (13.8)	13 (28.3)	7.773	0.021
ST-segment (%)	19 (10.4)	18 (19.1)	5 (10.9)	4.435	0.109
CK-MB increase (%)	73 (39.9)	50 (53.2)	26 (56.5)	6.752	0.034
Troponin rise (%)	0.04 (0.01, 0.25)	0.03 (0.01, 0.45)	0.05 (0.02, 0.38)	2.028	0.363
Cardiac arrest (%)	1 (0.5)	2 (2.1)	0 (0.0)	1.777	0.538
In-hospital PCI (%)	88 (48.1)	37 (39.4)	20 (43.5)	1.955	0.376
MACEs (%)	10 (5.5)	17 (18.1)	14 (30.4)	24.160	<0.001
Deaths (%)	0 (0.0)	3 (3.2)	6 (13.0)	20.000	<0.001
Non-fatal re-infarction (%)	6 (3.3)	11 (11.7)	7 (15.2)	11.135	0.004

*DC, deceleration capacity; STEMI, ST segment Elevation Myocardial Infarction; NSTE-ACS, non-ST-elevation acute coronary syndromes; SBP, Systolic blood pressure; DBP, Diastolic blood pressure; eGFR, glomerular filtration rate; NLR, neutrophil-to-lymphocyte ratio; PLT, platelet count; PLR, platelet-to-lymphocyte ratio; TG, triglycerides; TC, total cholesterol; HDL-C, high-density lipoprotein cholesterol; LDL-C, low-density lipoprotein cholesterol; Apo A1, Apolipoprotein A1; Apo B, Apolipoprotein B; Lp (a), lipoprotein a; MI, myocardial infarction; hs-CRP, high-sensitivity C-reactive protein; CK-MB, creatine kinase-MB; SDNN, standard deviation of all normal sinus RR intervals; SDANN, standard deviation average of normal-to-normal (NN) intervals; pNN50, percentage of the number of times that the difference between adjacent normal RR intervals >50 ms in the total number of NN intervals; rMSSD, root mean square successive difference; HF, high-frequency power; LF, low-frequency power; LF/HF, low-frequency/high-frequency ratio; MACEs, major adverse cardiac events*.

### The Relationship Between DC and MACEs

**Figure 2:**
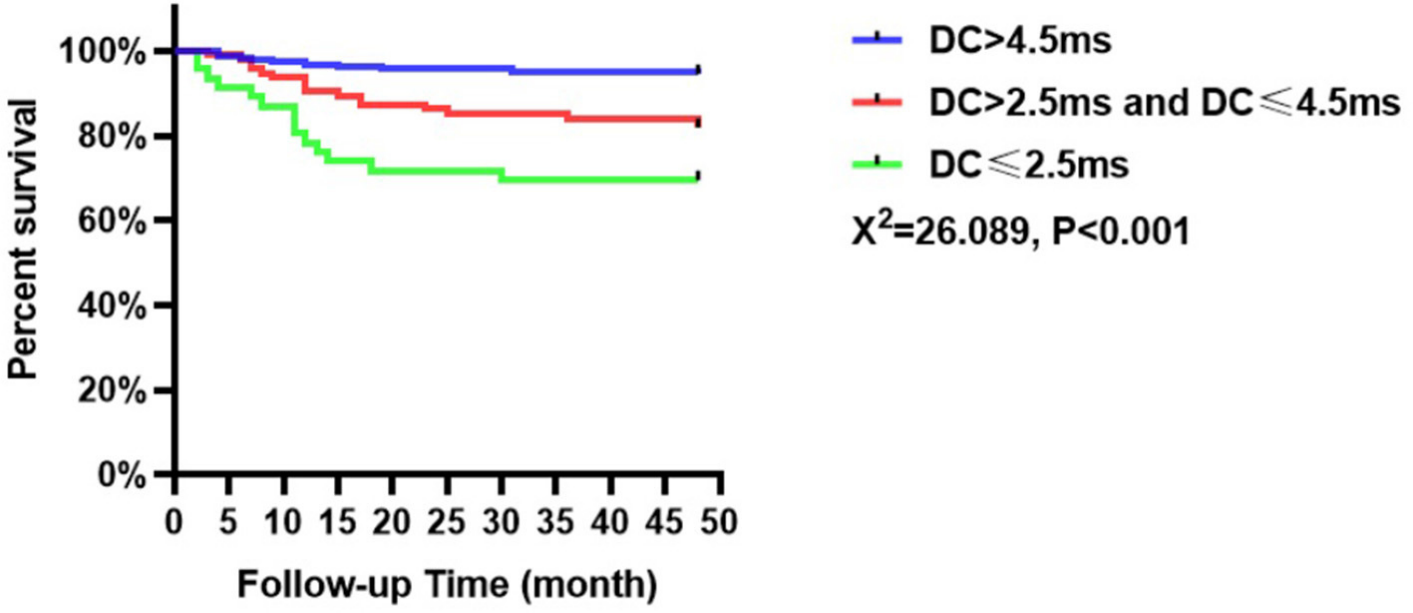
Cumulative survival of MACEs in patients with ACS based on the DC value.

The incidence of MACEs among the patients with ACS in this study was collected over an average follow-up of 43.78 months. Forty-one patients experienced MACEs, including 10 patients in the low-risk group (5.5%, *n* = 183), 17 patients in the intermediate-risk group (18.1%, *n* = 94), and 14 patients in the high-risk group (30.4%, *n* = 46). Kaplan–Meier analysis indicated that the incidence of MACEs was significantly different among patients with ACS based on DC values (χ^2^ = 26.089, *p* < 0.001, Figure 2). Besides patients in the high-risk group had a higher incidence of MACEs than those in the intermediate-risk and low-risk groups. In addition, those with intermediate risk were more susceptible to MACEs than those with low risk (*p* < 0.05).

### Predictors of MACEs

Univariate Cox analysis showed that history of MI, NSTE-ACS, neutrophil-to-lymphocyte ratio (NLR), platelet-to-lymphocyte ratio (PLR), creatinine, lipoprotein a [Lp (a)], DC, and GRACE score (all *p* < 0.05) were potential predictors of MACEs in patients with ACS (Table 2). Multivariate Cox analysis consistently showed that DC (HR: 0.885, 95% CI: 0.831–0.943, *p* < 0.001) and the GRACE score (HR: 1.020, 95% CI: 1.007–1.034, *p* = 0.002) were risk factors for MACEs at the final follow-up (Table 2).

**Table 2:** Predictors of the occurrence of major adverse cardiovascular events (MACEs) in patients with acute coronary syndrome (ACS): results of univariate and multivariate cox-regression analyses.

**Indicators**	**Univariate**				**Multivariate**			
	***P*** **-value**	**HR**	**95%CI**		***P*** **-value**	**HR**	**95%CI**	
Female (%)	0.547	0.817	0.423	1.577				
Age (years)	0.072	1.029	0.997	1.061				
Hypertension (%)	0.362	1.367	0.698	2.679				
Diabetes (%)	0.357	1.362	0.706	2.630				
Current smoker (%)	0.523	1.227	0.655	2.298				
History of drinking (%)	0.412	0.696	0.293	1.655				
Family history (%)	0.558	0.654	0.158	2.707				
History of MI (%)	0.001	2.922	1.513	5.643	0.172	1.672	0.800	3.496
Previous PCI (%)	0.118	1.649	0.880	3.088				
NSTE-ACS (%)	0.030	2.005	1.070	3.755	0.261	1.461	0.755	2.830
Neutrophil (× 10^9^/L)	0.204	1.016	0.991	1.042				
Lymphocyte (× 10^9^/L)	0.698	1.011	0.958	1.066				
NLR	0.001	1.053	1.021	1.087	0.191	1.052	0.975	1.135
PLT (× 10^9^/L)	0.753	0.999	0.994	1.005				
PLR	0.021	1.003	1.001	1.006	0.559	0.999	0.994	1.003
hs-CRP (mg/L)	0.509	1.004	0.992	1.016				
eGFR ml/(min1.73 m^2^)	0.138	0.988	0.973	1.004				
Creatinine (μmol/L)	<0.001	1.011	1.006	1.015	0.160	1.005	0.998	1.011
Uric acid (μmol/L)	0.302	1.001	0.999	1.004				
Glucose (mmol/L)	0.955	1.003	0.891	1.131				
TG (mmol/L)	0.353	0.845	0.592	1.206				
TC (mmol/L)	0.710	0.945	0.703	1.271				
HDL-C (mmol/L)	0.443	0.645	0.211	1.977				
LDL-C (mmol/L)	0.457	0.880	0.630	1.231				
Lp (a) (g/L)	0.027	1.001	1.000	1.002	0.083	1.001	1.000	1.002
Average heart rate (bpm)	0.760	1.005	0.973	1.038				
SDNN (ms)	0.138	0.992	0.982	1.003				
SDANN (ms)	0.993	0.999	0.992	1.008				
rMSSD (ms)	0.566	0.997	0.985	1.008				
Pnn50	0.551	0.988	0.950	1.028				
LF (ms^2^)	0.057	0.998	0.996	1.001				
HF (ms^2^)	0.708	1.001	0.999	1.002				
LF/HF	0.074	0.750	0.547	1.029				
DC (ms)	<0.001	0.876	0.832	0.923	<0.001	0.885	0.831	0.943
GRACE score	<0.001	1.033	1.022	1.044	0.002	1.020	1.007	1.034
SBP (mmHg)	0.159	1.011	0.996	1.027				
DBP (mmHg)	0.566	1.007	0.982	1.033				
Aspirin (%)	0.225	0.633	0.302	1.325			
Clopidogrel (%)	0.871	1.052	0.570	1.941				
Ticagrelor (%)	0.365	0.518	0.125	2.147				
Statins (%)	0.241	0.539	0.192	1.513				
β-blocker (%)	0.169	1.563	0.828	2.950				
ACEI (%)	0.576	1.246	0.576	2.698				
ARB (%)	0.264	1.467	0.749	2.875				
CCB (%)	0.137	1.611	0.860	3.017				
In-hospital PCI (%)	0.904	0.963	0.519	1.784				
The number of stent	0.574	0.895	0.608	1.318				

*NSTE-ACS, non-ST-elevation acute coronary syndromes; SBP, Systolic blood pressure; DBP, Diastolic blood pressure; eGFR, glomerular filtration rate; NLR, neutrophil-to-lymphocyte ratio; PLT, platelet count; PLR, platelet-to-lymphocyte ratio; TG, triglycerides; TC, total cholesterol; HDL-C, high-density lipoprotein cholesterol; LDL-C, low-density lipoprotein cholesterol; Apo A1, Apolipoprotein A1; Apo B, Apolipoprotein B; Lp (a), lipoprotein a; MI, myocardial infarction; hs-CRP, high-sensitivity C-reactive protein; CK-MB, creatine kinase-MB; SDNN, standard deviation of all normal sinus RR intervals; SDANN, standard deviation average of normal-to-normal (NN) intervals; pNN50, percentage of the number of times that the difference between adjacent normal RR intervals >50 ms in the total number of NN intervals; rMSSD, root mean square successive difference; HF, high-frequency power; LF, low-frequency power; LF/HF, low-frequency/high-frequency ratio; DC, deceleration capacity; ACEI, angiotensin converting enzyme inhibitor; ARB, angiotensin receptor blockers; CCB, calcium ion channel blockers; PCI, Percutaneous Transluminal Coronary Intervention*.

Moreover, subgroup analysis was based on STEMI and NSTE-ACS patients. Univariate Cox analysis showed that PLR, NLR, high-sensitivity C-reactive protein (hs-CRP), DC, and GRACE score (all *p* < 0.05) were predictors of MACEs for all of the evaluated STEMI patients, as shown in Table 3. Furthermore, for all of the evaluated STEMI patients, independent influencing factors for the incidence of MACEs included DC (HR: 0.901, 95% CI: 0.828–0.981, *p* = 0.016) and the GRACE score (HR: 1.024, 95% CI: 1.001–1.048, *p* = 0.043) according to multivariate Cox analysis (Table 3).

**Table 3:** Predictors of the occurrence of MACEs in patients with ST- segment Elevation Myocardial Infarction (STEMI): results of univariate and multivariate Cox-regression analyses.

**Indicators**	**Univariate**				**Multivariate**			
	***P*** **-value**	**HR**	**95% CI**		***P*** **-value**	**HR**	**95% CI**	
Female (%)	0.498	0.676	0.218	2.097				
Age (years)	0.094	1.043	0.993	1.096				
Hypertension (%)	0.463	0.691	0.257	1.855				
Diabetes (%)	0.066	2.523	0.939	6.776				
Current smoker (%)	0.233	1.824	0.679	4.899				
History of drinking (%)	0.540	0.676	0.193	2.371				
Family history (%)	0.745	0.714	0.094	5.409				
Past history of MI	0.162	2.242	0.723	6.954				
Previous PCI (%)	0.148	2.073	0.772	5.566				
Neutrophil (× 10^9^/L)	0.807	0.981	0.838	1.148				
Lymphocyte (× 10^9^/L)	0.087	0.433	0.166	1.131				
NLR	0.046	1.117	1.002	1.246	0.759	0.959	0.735	1.251
PLT (× 109/L)	0.795	1.001	0.993	1.010				
PLR	0.013	1.006	1.001	1.010	0.574	1.003	0.992	1.015
hs-CRP (mg/L)	0.043	1.018	1.001	1.035	0.622	1.006	0.982	1.032
eGFR ml / (min1.73 m^2^)	0.728	0.995	0.966	1.024				
Creatinine (μmol/L)	0.369	1.005	0.994	1.016				
Uric acid (μmol/L)	0.324	1.002	0.998	1.006				
Glucose (mmol/L)	0.604	1.047	0.879	1.248				
TG (mmol/L)	0.156	0.569	0.261	1.240				
TC (mmol/L)	0.338	0.788	0.483	1.284				
HDL-C (mmol/L)	0.112	0.166	0.018	1.518				
LDL-C (mmol/L)	0.436	0.818	0.493	1.357				
Lp (a) (g/L)	0.145	1.001	0.999	1.003				
Average heart rate (bpm)	0.620	0.985	0.929	1.045				
SDNN (ms)	0.269	0.990	0.973	1.008				
SDANN (ms)	0.315	0.991	0.975	1.008				
rMSSD (ms)	0.426	0.992	0.972	1.012				
Pnn50	0.374	1.022	0.974	1.073				
LF (ms^2^)	0.153	0.998	0.994	1.001				
HF (ms^2^)	0.064	1.002	0.999	1.003				
LF/HF	0.145	0.698	0.429	1.133				
DC (ms)	0.003	0.888	0.820	0.961	0.016	0.901	0.828	0.981
GRACE score	0.002	1.031	1.011	1.052	0.043	1.024	1.001	1.048
SBP (mmHg)	0.466	0.990	0.965	1.016				
DBP (mmHg)	0.085	0.958	0.913	1.006				
Aspirin (%)	0.540	0.629	0.143	2.768				
Clopidogrel (%)	0.206	1.977	0.687	5.690				
Ticagrelor (%)	0.970	0.962	0.127	7.284				
Statins (%)	0.197	0.377	0.086	1.659				
β-blocker (%)	0.488	0.699	0.254	1.924				
ACEI (%)	0.634	1.357	0.387	4.762				
ARB (%)	0.419	1.594	0.514	4.942				
CCB (%)	0.841	1.123	0.362	3.483				
In-hospital PCI (%)	0.142	2.206	0.766	6.351				
The number of stent	0.510	1.181	0.720	1.938				

Subsequent univariate Cox analysis further indicated that previous MI, NLR, creatinine, DC, GRACE score, and systolic blood pressure (SBP) (all *p* < 0.05) were potential predictors of MACEs among patients with NSTE-ACS, as shown in Table 4. For all of the evaluated NSTE-ACS patients, multivariate Cox analysis indicated that NLR (HR: 1.041, 95% CI: 1.000–1.084, *p* = 0.048), creatinine (HR: 1.013, 95% CI: 1.003–1.024, *p* = 0.011), DC (HR: 0.894, 95% CI: 0.811–0.986, *p* = 0.025), GRACE score (HR: 1.019, 95% CI: 1.002–1.036, *p* = 0.024), and SBP (HR: 1.034, 95% CI: 1.015–1.053, *p* < 0.001) were independent predictors for MACEs (Table 4).

**Table 4:** Predictors of the occurrence of MACEs in patients with Non-ST segment elevation myocardial infarction/ unstable angina (NSTEMI/UA): results of univariate and multivariate Cox-regression analyses.

**Indicators**	**Univariate**				**Multivariate**			
	***P*** **-value**	**HR**	**95%CI**		***P*** **-value**	**HR**	**95%CI**	
Female (%)	0.652	0.829	0.366	1.875				
Age (years)	0.528	1.013	0.973	1.055				
Hypertension (%)	0.082	2.386	0.895	6.359				
Diabetes (%)	0.687	0.828	0.331	2.073				
Current smoker (%)	0.740	1.159	0.484	2.775				
History of drinking (%)	0.940	0.955	0.286	3.190				
Family history (%)	0.719	0.692	0.094	5.117				
Past history of MI	0.005	3.243	1.432	7.344	0.159	1.937	0.772	4.858
Previous PCI (%)	0.428	1.391	0.615	3.149				
Neutrophil (× 10^9^/L)	0.311	1.013	0.988	1.039				
Lymphocyte (× 10^9^/L)	0.698	1.010	0.960	1.063				
NLR	0.024	1.042	1.005	1.079	0.048	1.041	1.000	1.084
PLT (× 10^9^/L)	0.648	0.998	0.991	1.006				
PLR	0.381	1.002	0.998	1.005				
hs-CRP (mg/L)	0.630	0.994	0.969	1.019				
eGFR	0.251	0.989	0.970	1.008				
Creatinine (μmol/L)	<0.001	1.021	1.013	1.030	0.011	1.013	1.003	1.024
Uric acid (μmol/L)	0.600	1.001	0.998	1.004				
Glucose (mmol/L)	0.717	0.971	0.829	1.138				
TG (mmol/L)	0.886	1.029	0.697	1.519				
TC (mmol/L)	0.493	1.156	0.764	1.751				
HDL-C (mmol/L)	0.666	1.341	0.354	5.084				
LDL-C (mmol/L)	0.926	1.024	0.625	1.677				
Lp (a) (g/L)	0.092	1.001	0.999	1.003				
Average heart rate	0.579	1.011	0.973	1.051				
SDNN (ms)	0.424	0.995	0.982	1.008				
SDANN (ms)	0.904	0.999	0.988	1.011				
rMSSD (ms)	0.628	1.004	0.988	1.020				
Pnn50	0.177	0.958	0.900	1.020				
LF (ms2)	0.241	0.999	0.997	1.001				
HF (ms2)	0.408	0.999	0.997	1.001				
LF/HF	0.715	0.926	0.612	1.400				
DC (ms)	<0.001	0.866	0.803	0.934	0.025	0.894	0.811	0.986
GRACE score	<0.001	1.031	1.017	1.045	0.024	1.019	1.002	1.036
SBP (mmHg)	0.014	1.024	1.005	1.044	<0.001	1.034	1.015	1.053
DBP (mmHg)	0.075	1.027	0.997	1.057				
Aspirin (%)	0.640	0.812	0.339	1.944				
Clopidogrel (%)	0.632	0.819	0.362	1.853				
Ticagrelor (%)	0.238	0.300	0.041	2.215				
Statins (%)	0.673	0.733	0.173	3.108				
β-blocker (%)	0.061	2.552	0.957	6.800				
ACEI (%)	0.849	1.100	0.413	2.931				
ARB (%)	0.657	1.210	0.522	2.803				
CCB (%)	0.202	1.666	0.760	3.652				
In-hospital PCI (%)	0.304	0.632	0.264	1.514				
The number of stent	0.319	0.729	0.392	1.357				

To assess whether models that included the GRACE score combined with DC or LF/HF presented a significantly better fit than those limited to the GRACE score alone, we compared nested models using the likelihood-ratio test. Our results demonstrated that the addition of DC (χ^2^ = 9.227, df = 1, *p* < 0.001) significantly enriched the predictive power of the existing model including the GRACE score to predict the incidence of MACEs (Table 5). In addition, the inclusion of LF/HF (χ^2^ = 0.329, df = 1, *p* = 0.416) did not optimize the model fit.

**Table 5:** Akaike's information criteria and likelihood ratio test to determine the best fitting model for prediction MACEs.

**Akaike's information criteria**							**Likelihood ratio test**		
**Model**	**AICc**	**δAICc**	**Relative likelihood**	**wi**	**wj/wi**	**Model**	***χ^2^***	**Df**	***P*** **-value**
GRACE score	781.44	5.82	0.15	0.14	4.18	GRACE score			
GRACE + LF/HF	776.35	4.29	0.21	0.20	5.35	GRACE+LF/HF	0.329	1	0.416
GRACE + DC	743.28	1.15	0.83	0.79	21.73	GRACE+DC	9.227	1	<0.001

The model including the GRACE score and DC had the lowest AICc and the highest Akaike's weight compared to the other two models, GRACE score alone and GRACE score with LF/HF (Table 5).

**Figure 3:**
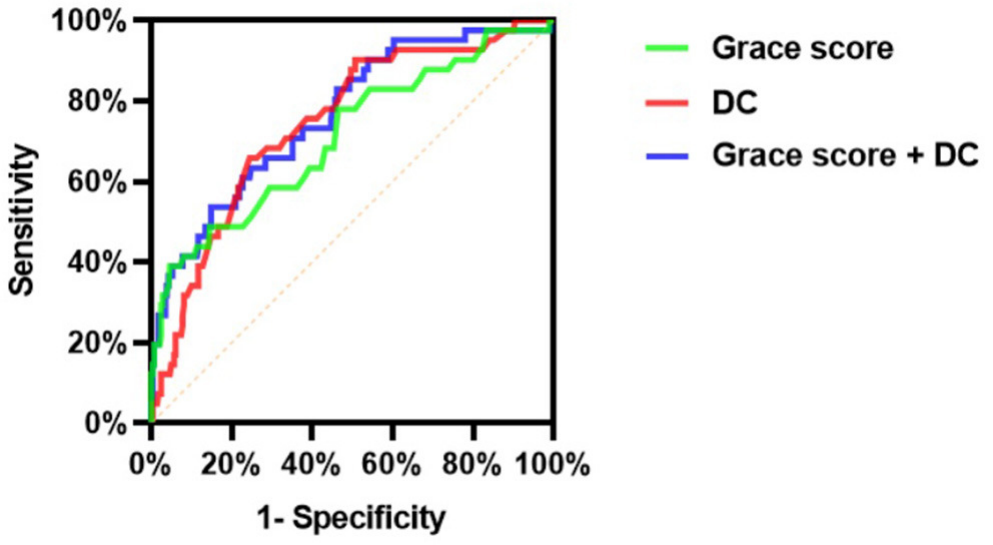
Comparison of discrimination and reclassification abilities of predictive models for MACEs (Model 1: Grace score; Model 2: DC; Model 3: Grace score + DC).

DC, but not LF/HF, combined with the GRACE score could improve the net reclassification of the updated model in predicting MACEs at the last follow-up date (Table 6, Central illustration). Employing continuous NRI (NRI^>0^), DC enhanced reclassification by 7.3% for patients with MACEs and by 12.8% for patients without MACEs, demonstrating a significant overall improvement in net reclassification (NRI 0.200, *p* = 0.003). Entering DC into a logistic regression model including the GRACE score appeared to predict a lower risk of MACEs than the GRACE score alone in both the MACE and MACE-free survival groups. The addition of LF/HF did not improve reclassification (NRI 0.04, *p* = 0.573). The addition of DC, but not LF/HF to the established model including the GRACE score promoted integrated discrimination, as evident in Table 6, Figure 3. Moreover, our results generated an IDI of 1.04%, *p* < 0.001.

**Table 6:** Net reclassification improvement for model improvement with the addition of DC or LF/HF to GRACE.

	**GRACE vs. GRACE + DC**				**GRACE vs. GRACE + LF/HF**			
	**NRIe**	**NRIne**	**Total**	***P*** **-value**	**NRIe**	**NRIne**	**Total**	***P*** **-value**
UP	22	123	145		20	132	152	
DWN	19	159	178		21	150	171	
Total	41	282	323		41	282	323	
NRI^>0^	0.073	0.128	0.200	0.003	−0.024	0.064	0.040	0.573
	**IDle**	**IDIne**	**Total**	***P*** **-value**	**IDle**	**IDIne**	**Total**	***P*** **-value**
Final	0.0089	0.0015	0.0104	<0.001	0.0003	0.0001	0.0004	0.422

**Central illlustration:**
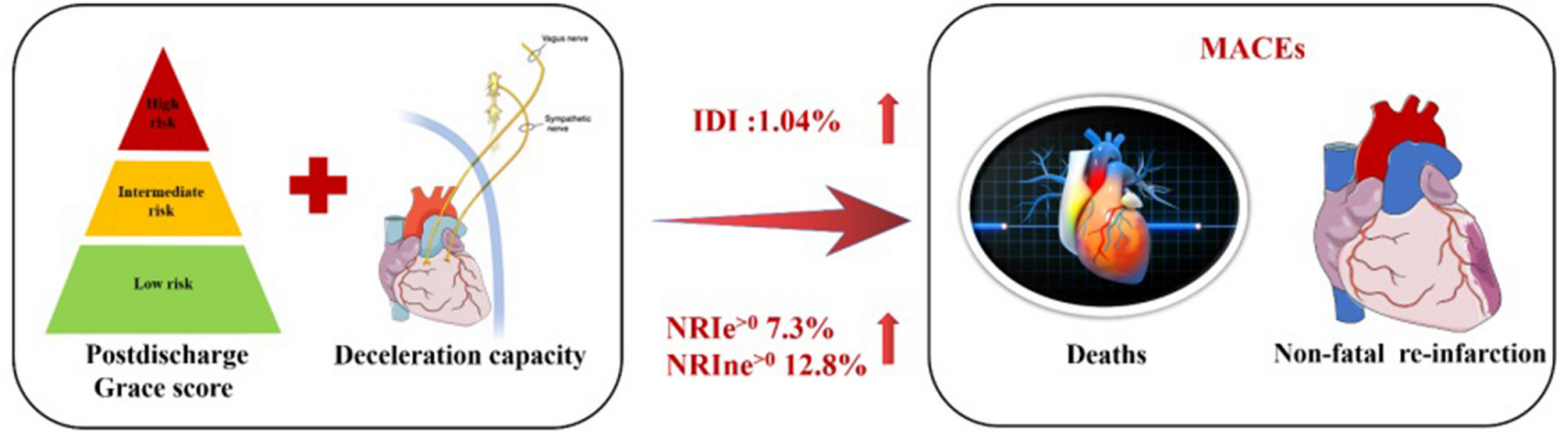
Combined efficacies of DC and postdischarge GRACE score for risk stratification in patients with ACS.

As presented in Figure 3, the c-statistic was 0.711 (95% CI 0.619–0.804, *p* < 0.001) for model 1 including the GRACE score only and 0.746 (95% CI 0.668–0.824, *p* < 0.001) for model 2 containing DC only. However, it was 0.765 (95% CI 0.686–0.844, *p* < 0.001) for the model including the GRACE score and DC (Table 7). For the prediction of MACEs, the positive c-statistic of the combined GRACE score was significantly improved in model 3 (AUC: 0.765; c-statistic: 0.783; 95% CI: 0.686–0.844; *p* < 0.001).

**Table 7:** ROC analysis comparing the predictive efficacies of related variables for the incidence of MACEs during follow up.

**Model**	**AUC**	**SE**	***P*** **-value**	**95%CI**	**Cut-off**	**Sensitivity**	**Specificity**	**Youden index**	**C-statistic**
Model 1	0.711	0.047	<0.001	0.619–0.804	116.5	48.8	85.5	0.343	0.726
Model 2	0.746	0.040	<0.001	0.668–0.824	3.51	53.7	85.1	0.388	0.759
Model 3	0.765	0.040	<0.001	0.686–0.844	-	65.9	75.5	0.414	0.783

*Model 1, Grace score; Model 2, DC; Model 3, Grace score + DC*.

## Discussion

Our studies now demonstrate that for ACS patients, wearable monitoring of cardiac ANS-related modulations by means of 24-h DC yields prognostic information beyond the known risk predictors. DC significantly optimizes risk stratification by the GRACE score concerning the prediction of MACEs during long-term follow-up. The prediction model including the postdischarge GRACE score and DC provided incremental prognostic information for long-term cohorts with established ACS. Furthermore, adding DC, rather than LF/HF, to the GRACE score could effectively improve the ability and accuracy of the GRACE score alone to predict MACEs after ACS.

Accumulating evidence supports the notion that the GRACE score provides valuable and independent prognostic information for ACS and enriches reliable risk stratification for identifying whether performing early PCI will benefit patients with ACS (3, 4, 19). In addition, previous study indicated that the GRACE score could predict short-term and long-term prognosis for ACS patients (20, 21). Although it is well established that the strong and effective prognostic value of the GRACE score has been confirmed by much evidence (3, 4, 19–21), early risk stratification remains urgently needed for further optimization, especially for low-risk patients with ACS. Therefore, previous studies have recently explored ways to improve the predictability of the prognostic GRACE score, including adding NT-pro-BNP (22), 2-h postload plasma glucose (23), plasma glucose blood inflammation-related indicators (24), plasma myeloperoxidase and trimethylamine N-oxide (25), serum acid uric acid (26) and nutritional risk index (27) to the GRACE score. The findings indicate that blood biochemical indexes and biomarkers provide incremental prognostic information for the predictive capacity of the GRACE score-based prognostic models. However, few studies have specifically focused on non-invasive markers and the GRACE score together to assess the joint prognostic effect.

Notably, the routine detection index of 24-h DC has also been considered to be a useful means for the screening and surveillance of high-risk post-MI patients (8). Moreover, our data are consistent with the finding that autonomic nervous system (ANS) imbalance carries a high risk for acute adverse events (28–30). The difference in risk between STEMI and NSTE-ACS may be explained by the different pathogenesis of the two diseases (31, 32). Therefore, our available data further demonstrated that DC remains an effective predictor in STEMI or NSTE-ACS.

In addition, other non-invasive indicators for the assessment of cardiac autonomic nerve function include HRV (7) and heart rate turbulence (33). However, heart rate turbulence and HRV indirectly reflect ANS modulation due to their poor stability, which limits their application in clinical practice (34–36). Nevertheless, DC is not susceptible to external interference and can reflect parasympathetic activity. Thus, in our study, DC, rather than LF/HF, could effectively increase the predictive capability of the postdischarge GRACE score-based prognostic models. A retrospective study that enrolled 1,821 patients with suspected ACS indicated a positive and independent correlation between short-term DC and short-term mortality among patients with suspected ACS (9). However, another study focused on the association between short-term DC combined with the admission GRACE score and short-term mortality, rather than the potential association between the 24-h DC combined with postdischarge GRACE score and long-term MACEs, which previously limited our understanding of the insights into the potential association between integration of the 24-h DC and postdischarge GRACE score and long-term poor outcomes. In addition, we used the 24-h Holter recordings to estimate the 24-h DC, which has been used as a daily clinical indicator in our practice. Additionally, patient's restrictively selected in the current study to minimize the impact of confounding factors on DC. Furthermore, we found that DC combined with the postdischarge GRACE score may reflect the interactions between ANS imbalance and adverse events, which may better predict poor long-term ensuing episodes of ACS. Given the recent increasing interest in individualized therapy for risk assessment, we believe that DC provides early valuable information for lifestyle modifications and monitoring of patients with ACS.

Physiologically, the ANS plays a crucial role in maintaining and promoting cardiac physiological function (37, 38). Pathologically, increasing research has confirmed that the context of acute myocardial ischemia could trigger an organismal stress response, induce cardiac sympathetic hyperactivity and suppress vagal activity, subsequently leading to coronary constriction, especially culprit vessel vasoconstriction, thus accelerating focal ischemia and hypoxia and causing the deterioration of myocardial ischemia (34, 39). In addition, previous clinical and basic research has shown that the vagus nerve of the ANS is involved in the regulation of the inflammatory response (40, 41), and the potential link among the ANS, inflammation and coronary artery physiology was confirmed by our previous studies (42, 43). Furthermore, our data confirmed that injured cardiac autonomic nerves in the setting of myocardial ischemia subsequently developed an elevated risk of MACEs after ACS. Therefore, we believe that the combination of DC and the GRACE score could enhance risk discrimination and provide important incremental prognostic information for long-term follow-up after ACS.

## Study Limitations

First, due to the purely retrospective observational design with long-term follow-up, our results were almost inevitably affected by recall bias and lost follow-up (44, 45). Second, this study has a small sample size and likely suffered from a lack of power. Our findings should be validated with larger samples and prospective studies in the future. Third, our study did not include coronary physiology, which might improve the predictive power when combined with DC and postdischarge GRACE scores. Thus, many known confounding factors were eliminated, but there was no guarantee about other unknown confounding factors. Finally, because it was a purely observational study, whether individualized and comprehensive therapy based on DC-optimized risk models translates into better outcomes remains to be established. Finally, we included only patients wih sinusrhythm, and the influence of the non-sinus rhythm in patients with ACS remains to be seen.

## Conclusions

Our study indicates that wearable devices that automatically evaluate the cardiac ANS by means of the 24-h DC value tend to be a useful risk-stratified indicator for MACEs among ACS patients, regardless of the type of ACS. Moreover, DC further optimized the GRACE score, which has long been regarded as the gold standard for quantitative risk assessment after ACS, providing increased discriminatory ability and accuracy for prognostic information. Where applicable, we highlight that attention should be given to implementing DC as part of comprehensive cardiovascular evaluation and clinical decision-making to enable us to design individualized prognostic therapies.

## Data Availability Statement

The datasets used and/or analyzed during this study are available from the corresponding author on reasonable request. Requests to access these datasets should be directed to LY, lileiyu@whu.edu.cn.

## Ethics Statement

Ethical approval was not provided for this study on human participants because this was a retrospective observational study, the Renmin Hospital of Wuhan University Ethics Committee granted an exemption from requiring ethics approval and informed consent from eligible patients was waived. The Ethics Committee waived the requirement of written informed consent for participation.

## Author Contributions

LY and HJ: substantial contributions to conception and design, data acquisition, or data analysis, and interpretation. LS, CL, JS, QD, YiW, ZZ, FG, LZ, YuW, and WT: drafting the article or critically revising it for important intellectual content. SD, JW, and FY: final approval of the version to be published and agreement to be accountable for all aspects of the work in ensuring that questions related to the accuracy or integrity of the work are appropriately investigated and resolved. All authors contributed to the article and approved the submitted version.

## Funding

National Natural Science Foundation of China [81871486, 81970287, and 82100530].

## Conflict of Interest

The authors declare that the research was conducted in the absence of any commercial or financial relationships that could be construed as a potential conflict of interest.

## Publisher's Note

All claims expressed in this article are solely those of the authors and do not necessarily represent those of their affiliated organizations, or those of the publisher, the editors and the reviewers. Any product that may be evaluated in this article, or claim that may be made by its manufacturer, is not guaranteed or endorsed by the publisher.

## Abbreviations

GRACE: Global Registry of Acute Coronary Events
CABG: coronary artery bypass grafting
PCI: Percutaneous Transluminal Coronary Intervention
ACS: acute coronary syndromes
STEMI: ST segment Elevation Myocardial Infarction
NSTE-ACS: non-ST-elevation acute coronary syndromes
MI: myocardial infarction
SBP: Systolic blood pressure
DBP: Diastolic blood pressure
eGFR: glomerular filtration rate
NLR: neutrophil-to-lymphocyte ratio
PLT: platelet count
PLR: platelet-to-lymphocyte ratio
TG: triglycerides
TC: total cholesterol
HDL-C: high-density lipoprotein cholesterol
LDL-C: low-density lipoprotein cholesterol
Apo A1: Apolipoprotein A1
Apo B: Apolipoprotein B
Lp (a): lipoprotein a
hs-CRP: high-sensitivity C-reactive protein
CK-MB: creatine kinase-MB
SDNN: standard deviation of all normal sinus RR intervals
SDANN: standard deviation average of normal-to-normal (NN) intervals
pNN50: percentage of the number of times that the difference between adjacent normal RR intervals > 50 ms in the total number of NN intervals
rMSSD: root mean square successive difference
HF: high-frequency power
LF: low-frequency power
LF/HF: low-frequency/high-frequency ratio
DC: deceleration capacity
MACEs: major adverse cardiac events
AUC: receiver-operator characteristic curve
NRI: net reclassification index
IDI: integrated discrimination improvement
IQR: interquartile range.
